# Genistein enhances TLR3-mediated apoptosis and immune signaling in breast cancer cells

**DOI:** 10.1007/s12032-025-02856-5

**Published:** 2025-08-22

**Authors:** Suleyman Kaleli, Asuman Deveci Ozkan, Gamze Guney Eskiler, Kaan Furkan Hamarat, Rabia Rana Derlioglu, Sevinc Yanar, Ecir Ali Cakmak

**Affiliations:** 1 Department of Medical Biology, Faculty of Medicine, Sakarya University, Sakarya, Türkiye; 2 Faculty of Medicine, Sakarya University, Sakarya, Türkiye; 3 Department of Medical Biology, Institute of Health Science, Sakarya University, Sakarya, Türkiye; 4 Department of Histology and Embryology, Faculty of Medicine, Sakarya University, Sakarya, Türkiye; 5 Department of Medical Biology, Faculty of Medicine, Ankara Yildirim Beyazit University, Ankara, Türkiye

**Keywords:** Genistein, Breast cancer, Apoptosis, Anticancer effect, TLR3

## Abstract

Breast cancer is one of the most common malignant tumors globally and the second leading cause of cancer-related death in women. Toll-like receptors (TLR) constitute a family of transmembrane receptors playing a crucial role in innate immunity. TLR3 is a type of TLR that is activated following Poly (I:C) double-stranded RNA binding. TLR3 activation leads to tumor suppression, and TLR3 directly causes apoptotic effects in cancer cells. Genistein (GEN), a phytoestrogen found in soy, inhibits cellular proliferation, induces apoptosis, and arrests the cell cycle. Therefore, it is important to determine the roles of immunotherapeutic agents targeting TLR3 in cancer treatment. The study aimed to determine the anti-inflammatory effect of GEN on breast cancer cells for the first time. The anti-inflammatory effects of GEN on the TLR3 signaling pathway were evaluated using Annexin V and cell cycle analysis, immunofluorescence assay, acridine orange staining, Western blotting, and ELISA cytokine release level in MCF-7 (hormone-dependent) and MDA-MB-231 (triple negative) breast cancer cells. The GEN alone treatment increased apoptosis, cell cycle arrest, apoptotic cell morphology, and the expression of TLR3, IRF3, AP-1, and p-NF-kB proteins. Additionally, higher levels of INF-β and TNF-α in both cells compared to treatment with Poly I:C alone were detected. These effects were more pronounced in MCF-7 cells than in MDA-MB-231 cells. Stimulation of the TLR3 signaling pathway was enhanced in the presence of GEN, leading to increased apoptosis.

## Introduction

Breast cancer is a prevalent type of cancer and the second leading cause of cancer-related deaths in women worldwide [1]. Although the exact cause of breast cancer has not been fully determined, it is known that various factors contribute to the development of the disease. Gender, age, family history of breast cancer, early onset of menstrual cycle, prolonged total menstrual activity, radiation exposure, and lack of physical activity are all significant risk factors [2]. Today, parameters such as tumor size, grade, histological subtype, and lymph vascular and axillary lymph node invasion are known to be the most critical factors in determining breast cancer prognosis. However, the evaluation of estrogen receptor (ER), progesterone receptor (PR), and human epidermal growth factor receptor-2 (HER-2) status in the tumor cells is also helpful in classification. [3]. The development and metastasis of breast tumors are influenced by immune system cells and associated inflammatory mediators in the tumor microenvironment. [4]. The balance between antitumor and pro-tumor inflammation determines whether the tumor will progress or be controlled. In addition, inflammation induced during breast cancer treatment is thought to be responsible for treatment resistance and plays a role in treatment-related metastasis and recurrence [4]. In one study, patients who received combination anti-inflammatory therapy had better outcomes than those who received standard breast cancer treatment [5]. Toll-like receptors (TLRs) are a family of transmembrane receptors that play a crucial role in innate immunity. TLRs are a type of receptors that act as pattern recognition receptors (PRRs) in the innate immune system. These receptors recognize highly conserved structures such as pathogen-associated molecular patterns (PAMPs) like bacteria, viruses, fungi, and parasites, and they activate the immune system by triggering a series of intracellular signaling cascades [6]. TLRs are specific to their ligands, and TLR3 recognizes double-stranded RNA/Poly (I:C) [7]. TLR3 activation leads to tumor suppression. Additionally, TLR3 activation mediates apoptosis via the extrinsic pathway in various cancer types [8–10]. Studies on hepatocellular carcinoma, neuroblastoma [11], esophageal squamous cell carcinoma [12], and non-small cell lung cancer [13] have shown that TLR3 expression is associated with a better prognosis. Furthermore, based on our previous research, we found that TLR3, when activated by Poly I:C, enhanced the anti-inflammatory properties of nobiletin in prostate cancer [14]. It also acted as an agonist with chemotherapeutic drugs, increasing the apoptotic effect in cells [15].

Dietary factors are believed to have a significant impact on both the prevention and progression of breast cancer [16]. There is a suggestion that soy consumption decreases the incidence of breast cancer in East Asian countries, leading to the exploration of soy and genistein (GEN), a phytoestrogen present in soy, as a potential therapeutic agent in breast cancer [17]. Plant-derived phytoestrogens like GEN are known as selective modulators of estrogen receptors. Studies have shown a link between consuming GEN and a lower incidence of breast cancer [18]. In vitro, studies suggest that GEN exerts anticancer activity in breast cancer cells due to selectively triggering ERb, which suppresses Erα signaling [19]. Furthermore, studies have shown that GEN can inhibit cellular proliferation [20], angiogenesis, and metastasis [21], as well as trigger apoptosis and arrest the cell cycle in the G2/M phase [20].

Although the anticancer properties of GEN have been extensively studied, many research has primarily focused on its pro-apoptotic, anti-proliferative, and anti-inflammatory effects in various cancer cell types [19–21]. However, its potential role in modulating innate immune signaling pathways, particularly the TLR3 pathway, remains largely unexplored. In this study, we investigate for the first time the ability of GEN to enhance TLR3-mediated signaling in MCF-7 and MDA-MB-231 breast cancer cells, both alone and in combination with Poly I:C, a synthetic TLR3 agonist. This approach provides novel insight into the immunomodulatory potential of GEN and suggests the mechanism that has not yet been for breast cancer therapy.

## Materials and methods

### Cell culture and viability analysis

The study used two types of breast cancer cell lines: hormone-sensitive (MCF-7) and hormone-insensitive (MDA-MB-231). The MCF-7 and MDA-MB-231 cell lines were grown in DMEM (Dulbecco's Modified Eagle Medium) with 2 mM L-glutamine, 50 IU/mL penicillin, and 50 mg/mL streptomycin. The optimal cytotoxic concentration or concentrations for alone GEN and the non-cytotoxic optimal concentration for alone Poly I:C and GEN + Poly I:C combination treatment were assessed using the WST-1 viability assay. For this study, MCF-7 and MDA-MB-231 cells were seeded into 96-well cell culture plates at a density of 2 × 10^4^ cells per well. The cells were then treated with Poly I:C (at 5 and 10 µM) [14] and GEN (at 5, 10, 25, 50, and 100 µM) [22] and incubated for 24, 48, and 72 h. Following this, 10 µL of WST-1 dye was added to each well and incubated for 1 h at 37 °C. Measurements were then taken using an Elisa Reader in the 460–620 nm wavelength range. The viability of control cells was considered 100%, and the viability rates of the experimental cells were expressed as percentages.

### Annexin V analysis

To assess the apoptotic effect of GEN, Poly I:C, and GEN + Poly I:C combination on MCF-7 and MDA-MB-231 cells, 1 × 10^5^ cells were seeded into a 6-well plate. After treatment with GEN, Poly I:C, and GEN + Poly I:C combinations, the cells were incubated at 37 °C with 5% CO_2_ for 24 h. Following the incubation, the cells were treated with trypsin at 37 °C with 5% CO_2_ for 5 min, centrifuged at 1200 rpm for 5 min, and washed twice with Phosphate Buffer Saline (PBS), each wash lasting 5 min. Annexin V dye was added to each tube containing the suspended cell groups and incubated in the dark for 30 min at room temperature. After incubation, each tube was analyzed using the Muse™ Cell Analyzer (Merck Millipore, Germany), and all experiments were repeated three times.

### Cell cycle analysis

To study the impact of GEN, Poly I:C, and the combination of GEN and Poly I:C on cell cycle arrest in MCF-7 and MDA-MB-231 cells, 1 × 10^5^ cells were seeded into a 6-well plate. After treatment with GEN, Poly I:C, and GEN + Poly I:C combinations, the cells were then incubated at 37 °C with 5% CO_2_ for 24 h. Subsequently, the cells were removed, fixed with 70% cold ethanol at −20 °C for at least 3 h, and then stained with 200 μL of Muse® Cell Cycle Reagent for 30 min following the protocol of the Muse® Cell Cycle Kit (Merck Millipore, Germany). The stained cell pellets were incubated for 30 min at room temperature in the dark and then analyzed using the Muse® Cell Analyzer (Merck Millipore, Germany). All experiments were repeated three times.

### Acridine orange (AO) staining

To observe the apoptotic effect of GEN, Poly I:C, and GEN + Poly I:C combination on MCF-7 and MDA-MB-231 cells, 1 × 10^5^ cells were seeded into a 6-well plate. After treatment with GEN, Poly I:C, and GEN + Poly I:C combinations, the cells were then incubated at 37 °C with 5% CO_2_ for 24 h. Following the incubation, the cells were washed twice with PBS each for 5 min, fixed with 4% Paraformaldehyde (PFA) for 30 min, and the wells were washed twice with PBS each for 5 min. Subsequently, the staining procedure was performed using Acridine Orange (Sigma-Aldrich, USA) solution for 30 min at room temperature in the dark. The stained wells were washed twice with PBS each for 5 min and then imaged using the EVOS™FLoid (Life Technologies, USA) cell imaging system.

### Immunofluorescence (IF) analysis

To investigate the impact of GEN, Poly I:C, and the combination of GEN and Poly I:C on the cellular localization of TLR3, TRIF, IRF3, p-NF-kB, and AP1 proteins in MCF-7 and MDA-MB-231 cells, 1 × 10^5^ cells were seeded into 6-well plates. Following treatment with GEN, Poly I:C, and GEN + Poly I:C combinations, the cells were then incubated at 37 °C and 5% CO_2_ for 24 h. After incubation, the cells were fixed with 4% PFA for 30 min, permeabilized using PBS containing 0.1% Triton X-100, and blocked with PBS containing 1% BSA and 5% goat serum for 30 min. Subsequently, the fixed cells were incubated with primary antibodies for 3 h, then washed twice with PBS each for 5 min, and incubated with Alexa Fluor 488-conjugated secondary antibody for 1 h. Finally, the nucleus was stained with DAPI and visualized using the EVOS™FLoid (Life Technologies, USA) cell imaging system.

### Western blotting analysis

To determine the levels of TLR3 (sc-32232, Santa Cruz), TRIF (sc-514384, Santa Cruz), IRF3 (sc-33641, Santa Cruz), p-NF-kB (3033, Cell signaling), and AP1 (MA5-15,172; Thermo Scientific) proteins in MCF-7 and MDA-MB-231 cells with the combination of GEN, Poly I:C, and GEN + Poly I:C, 1 × 10^5^ cells were seeded into T75 flasks. After treatment with GEN, Poly I:C, and GEN + Poly I:C combinations, the cells were incubated at 37 °C and 5% CO_2_ for 24 h. For protein isolation, the cells were collected by scraping from the flask and centrifuged at 12,000 rpm for 15 min. The obtained pellet was resuspended with a cold lysis buffer for 15 min; the total protein amount was determined using a protein amount determination kit (Thermo Scientific). β-Actin protein was used as the housekeeping gene. From the obtained protein stock, an appropriate amount of protein was diluted for loading, mixed with sample loading buffer, and separated by SDS-PAGE (4% stacking gel; 10% separating gel). The proteins in the gel were transferred to the membrane by electro-transfer (dry blotting system, Bio-Rad). Following the western blotting method, the membrane was shaken in blocking solution (5% milk powder, 0.05% Tween 20, 1 × TBS) for 30 min and washed three times with washing solution (0.1% Tween 20, 1 × TBS) each for 5 min. After this process, the primary antibody incubated for overnight at 4 °C and then the secondary antibody [Goat anti-Mouse (31,430) and Rabbit IgG (31,460) (H + L) Secondary Antibody, HRP (Thermo Scientific)] was incubated for 2 h at room temperature specifically for the proteins. The signal was detected with the imaging device (Syngene GBox) using the ECL (enhanced chemiluminescence) kit (Thermo Scientific).

### Enzyme-linked immunosorbent Assay (ELISA)

To determine the effect of GEN, Poly I:C, and GEN + Poly I:C combination on INF-β and TNF-α levels in MCF-7 and MDA-MB-231 cells, 1 × 10^5^ cells were seeded into 6-well plates after treatment with GEN, Poly I:C, and GEN + Poly I:C combination the cells were incubated at 37 °C and 5% CO_2_ for 24 h. After incubation, the medium was taken, and INF-β (E-EL-H0085) and TNF-α (E-EL-H0109) were determined with the ELISA kit according to the procedure recommended by the company (Elabscience).

### Statistical analysis

Statistical analyses were performed using GraphPad (V9.0) software package programs. P values ​​less than 0.05 will be considered statistically significant. The intensities of the bands obtained from Western analysis will be determined using the Image J program. The normality of the data distribution was assessed using the Shapiro–Wilk test. Following confirmation of normal distribution, a two-way analysis of variance (ANOVA) was conducted to evaluate the effects of two independent variables. Post hoc comparisons between groups were performed using Tukey’s multiple comparison test.

### Effect of GEN, Poly I:C, and GEN + Poly I:C combination on cell growth

**Fig. 1:**
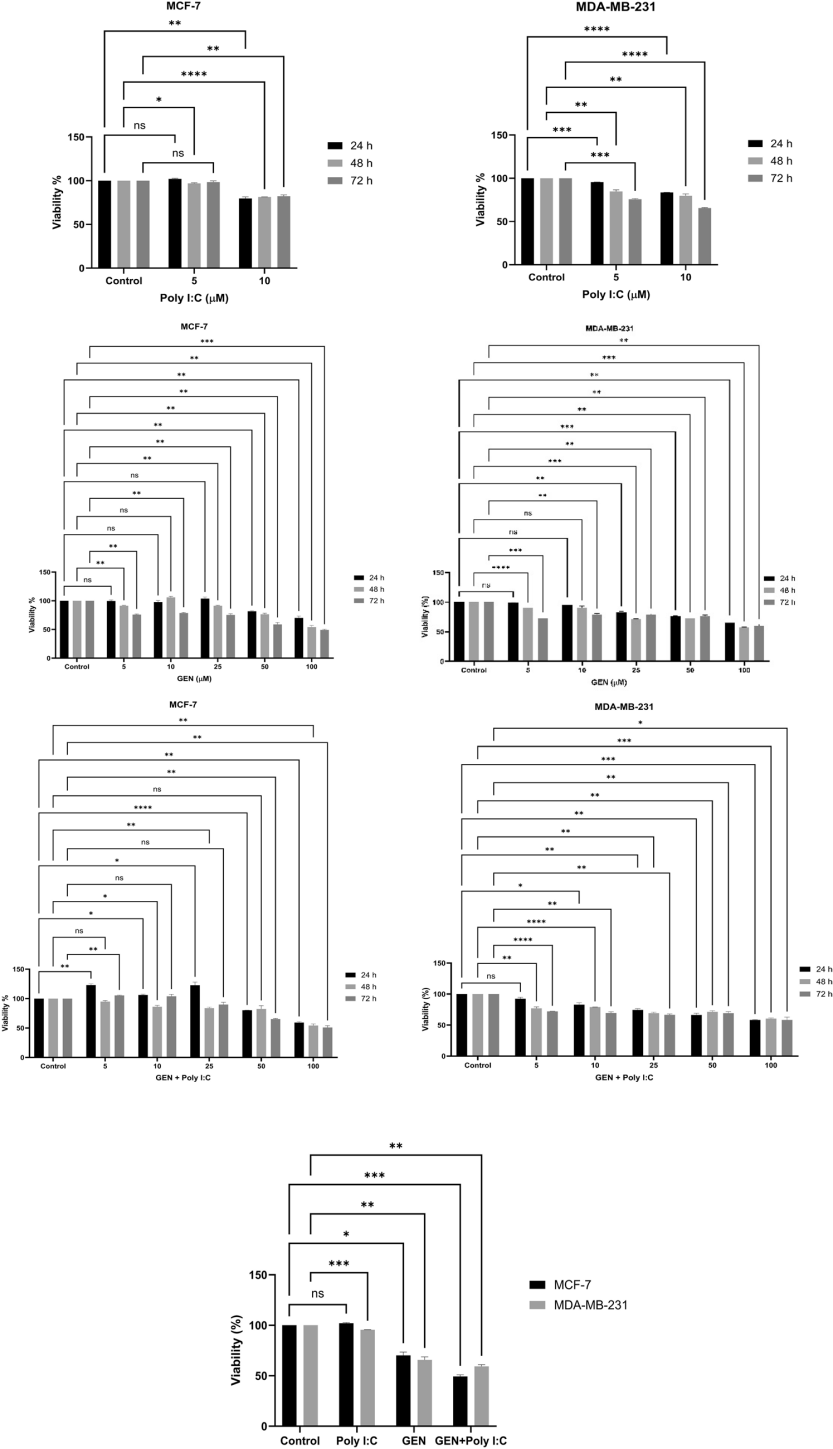
Effect of Poly I:C and GEN on the viability of MCF-7 and MDA-MB-231 breast cancer cells. (a) Cell viability of MCF-7 and MDA-MB-231 cells treated with Poly I:C (5 and 10 µM) for 24, 48, and 72 h, (b) Effect of GEN treatment (5, 10, 25, 50, and 100 µM) on the viability of MCF-7 and MDA-MB-231 cells at different time points (24, 48, and 72 h), (c) Combined effect of GEN (5, 10, 25, 50, and 100 µM) and 100 µM Poly I:C on cell viability at 24, 48, and 72 h, (d) Comparison of cell viability between 100 µM GEN treatment alone and 100 µM GEN + 5 µM Poly I:C combination in both cell lines. Data are presented as mean ± standard deviation (n = 3). Statistical significance is indicated by *p < 0.01, **p < 0.05, ***p < 0.001, and ****p < 0.0001 compared to the control group

The concentrations of GEN and Poly I:C, as well as the treatment durations (24, 48, and 72 h), were evaluated to identify conditions that affect cell viability in MCF-7 and MDA-MB-231 cells using WST-1 assays (Fig. 1). Poly I:C alone (5 and 10 µM) resulted in modest reductions in cell viability, suggesting limited cytotoxicity at these concentrations (Fig. 1A). Based on these results, 5 µM Poly I:C was selected for combination treatments to minimize toxicity while allowing potential TLR3 activation. GEN treatment led to a concentration- and time-dependent reduction in cell viability (Fig. 1B). GEN and 5 µM Poly I:C combination resulted in a slightly greater reduction in viability at higher GEN concentrations (especially 100 µM), particularly after 24 h (Fig. 1C). Although the observed effects of the combination were more pronounced than GEN alone, the differences were relatively modest. Thus, while the GEN + Poly I:C combination demonstrated enhanced cytotoxicity compared to individual treatments, the magnitude of this enhancement was limited. Thus, further studies are needed to confirm the biological significance and underlying mechanisms (Fig. 1D).

### Genistein exhibits inducible effect on apoptotic cell death

**Fig. 2:**
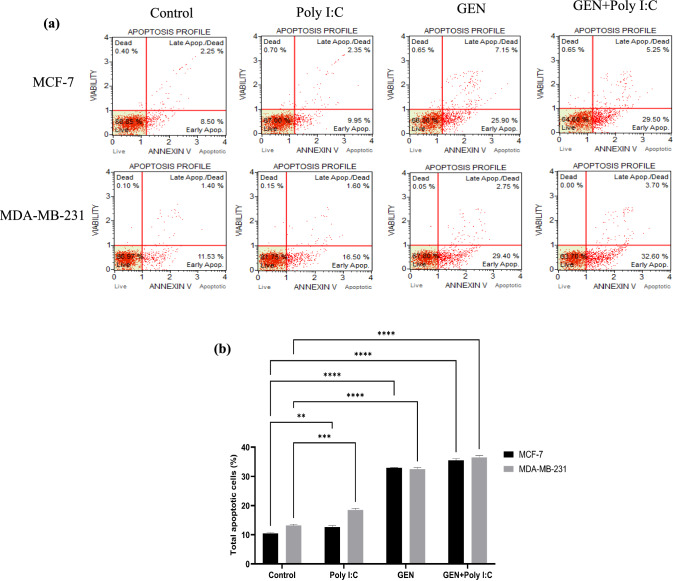
Induction of apoptosis in MCF-7 and MDA-MB-231 breast cancer cells following treatment with Poly I: C and GEN. (a) Representative flow cytometry dot plots showing apoptosis profiles of MCF-7 and MDA-MB-231 cells after treatment with control (untreated), Poly I:C, GEN, or GEN + Poly I:C. Apoptotic cells were assessed using Annexin V/propidium iodide (PI) staining. The lower-left quadrant represents live cells, the lower-right quadrant shows early apoptotic cells (Annexin V-positive/PI-negative), the upper-right quadrant represents late apoptotic or dead cells (Annexin V-positive/PI-positive), and the upper-left quadrant shows necrotic cells (PI-positive/Annexin V-negative). (b) Quantification of total apoptotic cells (early + late apoptosis) in MCF-7 and MDA-MB-231 cells under different treatment conditions. Data are presented as mean ± standard deviation (n = 3), and statistical significance is indicated by **p < 0.05, ***p < 0.001, and ****p < 0.0001 compared to the control group

Annexin V analysis was conducted to assess the apoptotic effects of GEN, Poly I:C, and their combination in MCF-7 and MDA-MB-231 cells (Fig. 2). In MCF-7 cells, the total apoptotic rate was 32.96 ± 0.09% following 100 µM GEN treatment, 12.61 ± 0.36% with 5 µM Poly I:C, and 35.53 ± 0.38% with the GEN + Poly I:C combination, compared to 10.43 ± 0.31% in the control group (p < 0.01). Similarly, in MDA-MB-231 cells, total apoptosis was 32.51 ± 0.36% upon GEN, 18.51 ± 0.41% upon Poly I:C, and 36.55 ± 0.46% upon the combination treatment, compared with the control (13.19 ± 0.26%) (p < 0.01). These results indicate that GEN induces apoptotic response in both cell lines, and the apoptotic effects of combination were more pronounced following combination treatment (Fig. 2).

### Acridine orange staining reveals morphological features of apoptosis

**Fig.3:**
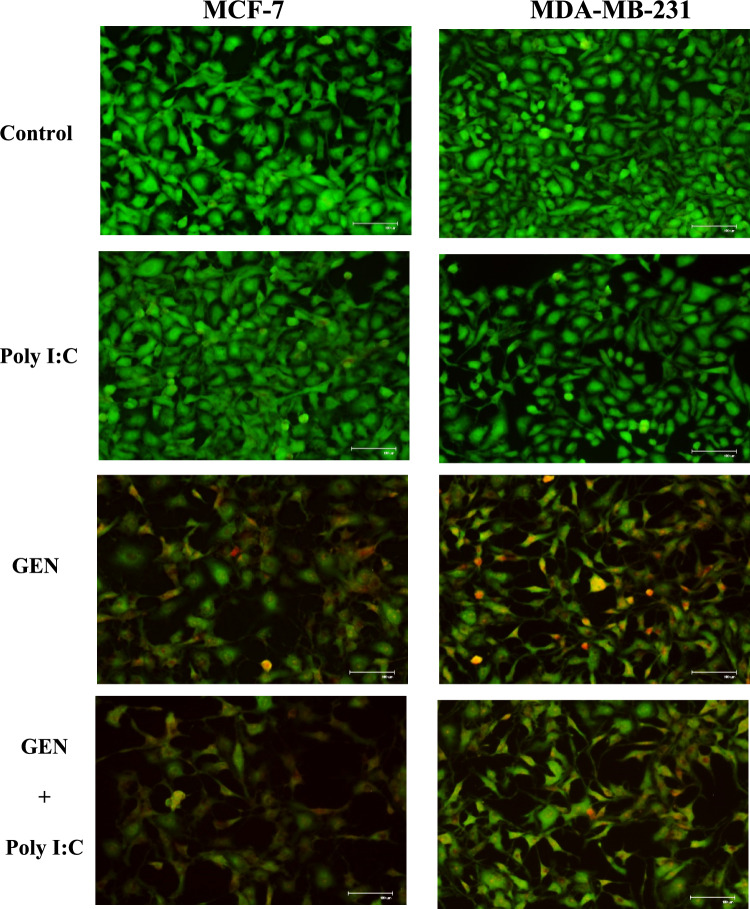
Effect of GEN and Poly I:C on MCF-7 and MDA-MB-231 breast cancer cell viability. Representative fluorescence microscopy images showed the morphology of MCF-7 (left) and MDA-MB-231 (right) cells under different treatment conditions. Scale bars were shown in each image

To qualitatively assess the apoptotic effects of GEN, Poly I:C, and their combination, acridine orange (AO) staining was performed on MCF-7 and MDA-MB-231 cells (Fig. 3). Cells treated with 100 µM GEN exhibited characteristic apoptotic features such as membrane blebbing and nuclear condensation. These morphological changes appeared slightly more pronounced in the combination treatment group (GEN + Poly I:C), particularly in MCF-7 cells. AO/PI staining indicated that GEN alone and GEN + Poly I:C treatments induced apoptotic death in two different breast cancer cells.

### Genistein induced cell cycle arrest at G2/M phase

**Fig. 4:**
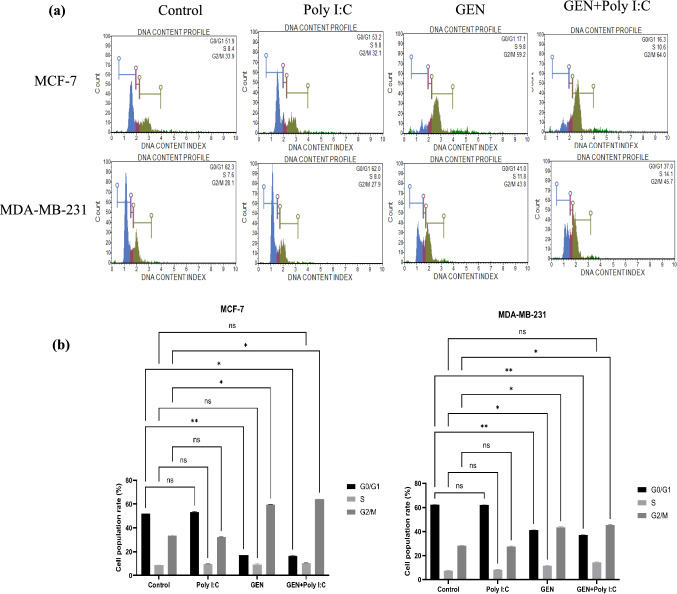
Cell cycle analysis of MCF-7 and MDA-MB-231 cells following treatment with Poly I:C, GEN or GEN + Poly I:C. (a) Representative DNA content histograms obtained by flow cytometry, showing cell cycle distribution (G0/G1, S, and G2/M phases), (b) Quantification of the G2/M phase percentage for MCF-7 and MDA-MB-231 cells under each treatment condition. Data are presented as the mean ± SD (n = 3). Statistical significance is indicated by *p < 0.01, and **p < 0.001 compared to the control group

The cell cycle analysis was performed to assess the effects of GEN, Poly I:C, and their combination on cell cycle distribution in MCF-7 and MDA-MB-231 cells (Fig. 4). A modest but statistically significant increase in G2/M phase accumulation was observed following GEN treatment in both cell lines. In MCF-7 cells, GEN treatment led to an increase in the G2/M population from 33.9% (control) to 58.9%, and 64.4% with the GEN + Poly I:C combination (p < 0.01). Similarly, in MDA-MB-231 cells, the G2/M phase population increased from 28.1% (control) to 44.0% with GEN, and to 45.4% with the combination treatment (p < 0.01). These results suggest that GEN, alone or in combination with Poly I:C, may contribute to G2/M phase arrest, particularly in MCF-7 cells.

### Genistein changes cellular localization of TLR3 pathway-related proteins

**Fig.5:**
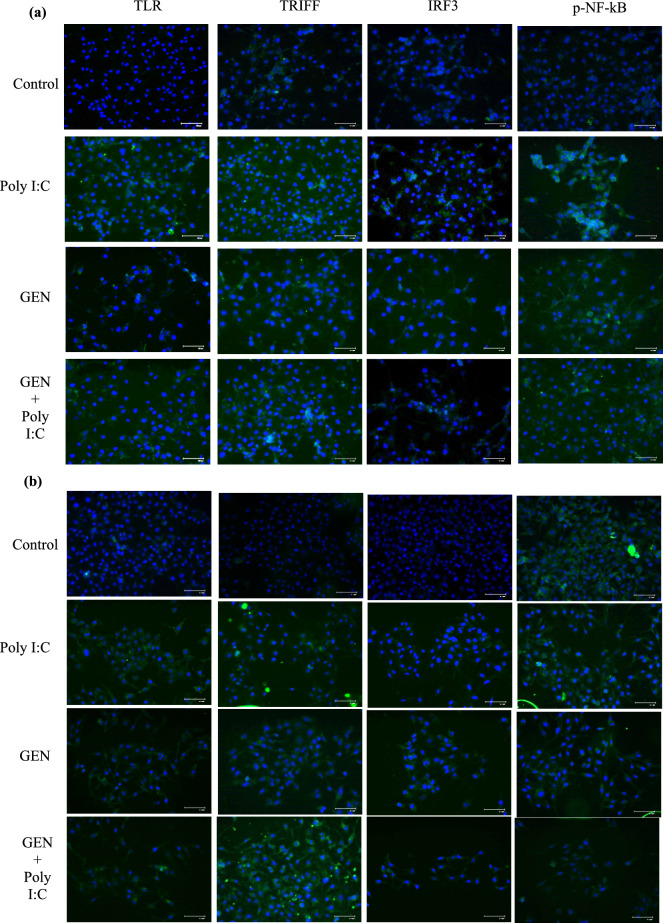
IF detection of TLR-3, TRIF, IRF3, and p-NF-kB in breast cancer cells treated with Poly I:C, GEN or GEN + Poly I:C (a) Representative images of MCF-7 and (b) MDA-MB-231 cells under each indicated treatment. TLR-3, TRIF, IRF3, and p-NF-kB are stained in green, while nuclei are counterstained with DAPI (blue). Scale bars are shown in each panel

Immunofluorescence analysis was performed to evaluate the effects of GEN, Poly I:C, and their combination on the cellular localization of TLR3 pathway-related proteins (TLR3, TRIF, IRF3, and p-NF-kB) in MCF-7 and MDA-MB-231 cells (Fig. 5). Compared to the control group, treatment with Poly I:C appeared to enhance cytoplasmic localization of TLR3 and p-NF-kB, along with a noticeable nuclear translocation of TRIF and IRF3 in both cell lines. Treatment with GEN alone showed relatively modest changes in localization of these proteins. However, the combination of GEN and Poly I:C seemed to further increase cytoplasmic expression of TLR3 and p-NF-kB and enhance nuclear translocation of TRIF and IRF3, particularly in MCF-7 cells. While these observations may suggest enhanced TLR3 pathway engagement under combination treatment, the extent and functional consequences of these localization changes require further validation through quantitative analysis and complementary assays.

### TLR3 pathway activation and cytokine induction by Poly I:C alone and in combination with GEN

**Fig.6:**
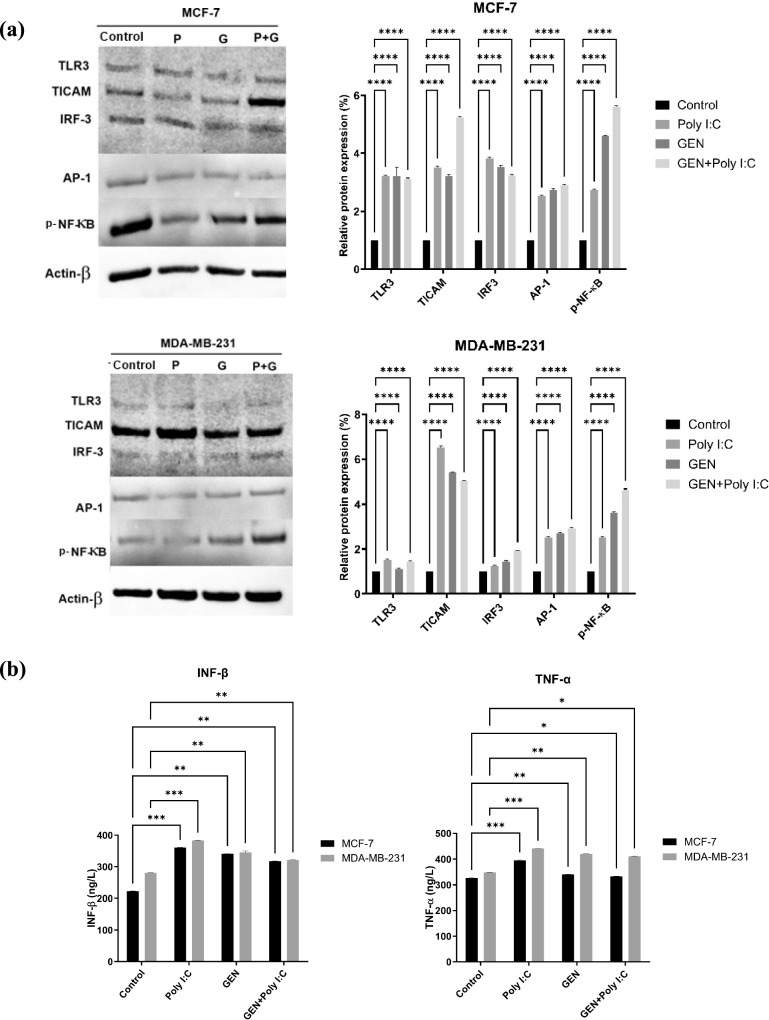
Western blot and ELISA analysis results. (a) Western blot analysis of TLR3, TICAM, IRF-3, AP-1, and p-NF-kB protein levels in MCF-7 and MDA-MB-231 breast cancer cells following treatment with Poly I:C (P), GEN (G) or GEN + Poly I:C (G + P). β-Actin served as an internal control. (b) Quantification of TNF-α and IFN-β secretion by MCF-7 and MDA-MB-231 cells following treatment with Poly I:C, GEN or GEN + Poly I:C, measured by ELISA. Data are presented as the mean ± SD (n = 3). Statistical significance is indicated by *p < 0.01, **p < 0.05, ***p < 0.001, and ****p < 0.0001 compared to the control group

The expression levels of TLR3 pathway-associated proteins (TLR3, TRIF, IRF3, AP-1, and p-NF-kB) were analyzed by Western blotting in MCF-7 and MDA-MB-231 cells following treatment with GEN, Poly I:C, and their combination (Fig. 6A). Compared to the control group, Poly I:C treatment was associated with increased expression of TLR3, IRF3, AP-1, and p-NF-kB proteins in both cell lines. The combination of GEN and Poly I:C resulted in higher expression levels than GEN treatment alone. This effect appeared more prominent in MCF-7 cells. In parallel, total levels of TNF-α and IFN-β proteins, measured by ELISA, were elevated in both cell lines following Poly I:C treatment, with a further increase observed upon GEN + Poly I:C treatment (Fig. 6B). The increases were generally more pronounced in MCF-7 cells compared to MDA-MB-231 cells. These findings suggest that GEN could modulate the expression of TLR3-related proteins and cytokines when used alone or in combination with Poly I:C.

## Discussion

TLR3 activation directly inhibits tumor growth in cancer cells. This occurs through the inhibition of cell proliferation and the induction of apoptotic cell death in various types of cancer cells, including breast [3], melanoma [23], prostate [15], and lung [13]. Studies have shown that targeting TLR3 activation to induce apoptosis could be a potential therapeutic approach in cancer treatment [24]. The idea that consuming GEN, a phytoestrogen found in soy, reduces the rate of breast cancer in East Asian countries has brought attention to phytoestrogens as potential therapeutic agents in breast cancer. It has been reported that GEN inhibits cellular proliferation, angiogenesis, and metastasis, as well as induces apoptosis and arrests the cell cycle in the G2/M phase. However, there is no literature study to determine the role of TLR signaling pathways in the anti-inflammatory effect of GEN. Therefore, this study aimed to determine the anti-inflammatory effect of GEN on breast cancer cells due to the activation of the TLR3 signaling pathway via Poly I:C stimulation. Additionally, the study evaluated the effect of GEN on the TLR3 signaling pathway at the protein level and determined cytokine release levels. Our study found that GEN increased apoptosis, cell cycle arrest, and apoptotic cell morphology in both cell types compared to the Poly I:C alone treatment and caused more apoptotic cell death in MCF-7 cells than in MDA-MB-231 cells upon combination treatment. Moreover, we observed that GEN + Poly I:C treatment in both MCF-7 and MDA-MB-231 cells significantly increased the expression of TLR3, IRF3, AP-1, and p-NF-kB proteins, as well as the amount of INF-β and TNF-α proteins. It was also determined that TLR3 signaling pathways were stimulated more in the presence of GEN in MCF-7 cells than in MDA-MB-231 cells and that TLR3 signaling pathway-dependent apoptosis was increased.

GEN exhibits numerous biomedical effects, such as antioxidation, antiproliferation, and tumoricidal activities [25]. More importantly, in vivo, in vitro, and in silico studies on its anticancer properties point to an important role of GEN as an antitumor molecule in various types of cancer [26]. Two significant reasons for the extensive research on GEN in recent years are the evidence of lower disease risk associated with its administration and the search for pharmacological drugs that affect growth factor signaling pathways in cells. Numerous previous studies have reported the arrest of the cell cycle and apoptosis in multiple cancer cell lines in vitro studies [27]. In studies examining the effects of GEN on prostate and head cancer cell lines, researchers found that GEN can stop cell cycle progression in the G2/M phase. This occurs because GEN decreases the expression of cyclin B, which suggests that it could be a potent regulator of cyclin B with potential applications in cancer prevention [28]. Furthermore, in a study focusing on the molecular effects of GEN on head cancer cells, GEN can halt cell cycle progression and induce cell death in a head cancer cell line by regulating p21WAF1 and Bax and by suppressing cyclin B1 and Bcl-2. In our study, we also observed cell cycle arrest in the G2/M phase in MCF-7 and MDA-MB-231 cells treated with GEN alone, which aligns with findings from previous research.

GEN exerts estrogen-like and, antiestrogenic and anticancer properties [29]. Due to its structural similarity to estrogen, GEN may exhibit activities that mimic estrogen in the body. It acts on estrogen receptors α and β through classical genomic mechanisms [30]. Supporting this information, this study found that GEN showed higher toxicity and increased apoptotic death in MCF-7 cells, which are ER + and hormone-sensitive, compared to ER- and hormone-insensitive MDA-MB-231 cells. In summary, based on preclinical and clinical evidence, GEN exhibits apparent dose-dependent breast cancer-preventive effects achieved through several different molecular pathways. Based on these findings, GEN could be a potent breast cancer-preventive agent.

TLR3 signaling is regulated by binding the adaptor protein TRIF, inducing the TIR domain of the TLR3 receptor. TLR3 leads to the activation of the transcription factors NF-κB and IRF3 and the synthesis of interferons at the end of the TLR3 signaling pathway [31]. TRIF also exhibits pro-apoptotic activity, suggesting that TLR3 signaling triggers apoptosis [32]. Type I interferon production is necessary for cell death induced by TLR3 agonists, but it is insufficient for apoptosis. NF-κB, p65, and extrinsic caspases are also required for TLR3-induced apoptosis [33]. Based on the results obtained from this study, we have demonstrated that GEN can be a potent stimulant for apoptotic cell death by activating these pathways. Furthermore, it has the potential to be a robust anti-inflammatory agent due to its ability to modulate the TLR3 signaling pathway, especially when combined with Poly I:C.

TLR stimulation using MyD88, TRIF, or both can induce apoptosis. This indicates that apoptosis can be activated independently by MyD88 and TRIF, and the combined signaling pathways initiated by these adaptor proteins may lead to apoptosis. The role of TLRs in cancer cells varies depending on the type and source of cancer. Therefore, more studies and evidence are needed for the clinical use of TLR agonists as therapeutic agents. It is essential to understand the molecular mechanism of TLR3-mediated apoptosis fully. Aside from death receptors, the signaling pathway initiated by TLR3 plays a significant role in regulating apoptosis and may have potential therapeutic benefits. Consequently, the role of TLRs in cancer has become of interest to researchers. Herbal agents with strong anticancer effects, such as GEN, have fewer side effects and may be more effective modulators in cancer cells. Further investigation into their effects on the TLR signaling pathways is necessary. Despite the statistically significant differences observed in cell viability and apoptosis assays, the magnitude of these effects was relatively modest. Therefore, the biological significance of the GEN and Poly I:C combination treatment should be interpreted with caution. Furthermore, the current study is limited to in vitro analysis using two breast cancer cell lines, and the results may not fully reflect the complexity of tumor behavior in vivo. Further studies involving primary cell cultures or in vivo models are needed to confirm these findings. Mechanistic insights into how TLR3 activation modulates GEN-induced apoptosis have not been explored and remain an important area for future investigation. Additional studies, including gene expression profiling, pathway analyses, and in vivo validation, are warranted to better understand the synergistic or additive effects observed.

One limitation of this study is the use of established cell lines, which may not fully replicate the complexity of primary tumor cells. Further studies involving primary cell cultures or in vivo models are needed to confirm these findings.

In conclusion, while GEN exhibits anticancer and anti-inflammatory properties in breast cancer treatment, the effects observed in this study, particularly in MCF-7 cells, were generally modest. Our findings suggest that GEN modestly enhance TLR3 signaling and may act as a weak TLR3 agonist. Further investigations are required to clarify the extent and significance of GEN’s interaction with Poly I:C, as well as its potential involvement in other TLR signaling pathways. Additionally, future studies should explore the underlying molecular mechanisms in more depth and across diverse cellular models to confirm and extend these preliminary findings.

## Data Availability

No datasets were generated or analyzed during the current study.
